# Consequences of Acute Presentations of Functional Neurological Disorders in Neuro‐Oncology Patients: Case Series and Systematic Review

**DOI:** 10.1002/brb3.71107

**Published:** 2025-11-30

**Authors:** Stuart C. Innes, Dorothy K. Joe, Katia Cikurel, José P. Lavrador, Francesco Vergani, Ranj Bhangoo, Keyoumars Ashkan, Gerald T. Finnerty

**Affiliations:** ^1^ Department of Basic and Clinical Neuroscience King's College London London UK; ^2^ Department of Neurology King's College Hospital NHS Foundation Trust London UK; ^3^ Department of Neurosurgery King's College Hospital NHS Foundation Trust London UK

**Keywords:** brain tumor, seizure, speech, stroke

## Abstract

**Introduction:**

New neurological symptoms in neuro‐oncology patients are usually attributed to the tumor or its treatment. A diagnosis of functional neurological disorder (FND) is often only considered when investigations do not reveal a cause and medical management fails. The consequences to neuro‐oncology patients of comorbid FND have not been elaborated.

**Methods:**

We performed a single‐center retrospective case study of adult neuro‐oncology patients with an intracranial tumor who presented acutely with FND and required expedited investigations. Data recorded were tumor type, investigations, adverse life events, medical interventions, and outcomes. This was combined with a systematic literature review.

**Results:**

Ten patients met our study criteria. Six had functional seizures, two had functional hemiparesis, and two had functional speech disorders. FND symptoms started prior to tumor diagnosis in three patients; between diagnosis and tumor treatment in three patients; and after treatment commenced in four patients. Two patients were thrombolyzed for a presumed stroke. Three patients had either their tumor surgery or chemoradiotherapy delayed. Diagnosis and management of FND enabled tumor treatment to restart. The systematic review identified 43 patients. Thirty‐nine had functional seizures, and four had motor FND. All FNDs except one started after tumor treatment commenced.

**Conclusion:**

Acute FND can occur at any stage of a brain tumor illness. The FND may lead to unnecessary medical interventions and can disrupt tumor treatment. Although acute FND improved with diagnosis and explanation, many neuro‐oncology patients require a multidisciplinary tumor–FND pathway to manage acute FND and avoid delays to tumor treatment.

## Graphical abstract

Functional neurological disorders (FNDs) can present acutely at any stage of a brain tumor illness and may result in inappropriate investigations and treatment. Improved awareness of this issue will facilitate earlier diagnosis of FND, encourage development of multidisciplinary pathways to treat FND, and prevent delays to tumor treatment.

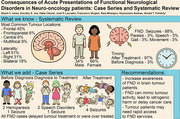

## 1 Introduction

People with brain and meningeal tumors report a wide variety of symptoms, such as seizures, focal neurological deficits, headache, and cognitive dysfunction (Taphoorn and Klein 2004; Weller et al. 2021). The symptoms usually reflect the tumor location or may be side effects from tumor treatment (Heimans and Taphoorn 2002; Kirby and Finnerty, 2021). There is increasing awareness, however, that people with neurological diseases not due to a tumor can develop new symptoms that are the result of a functional neurological disorder (FND) rather than a worsening of the original neurological disease (Carter 1955; Stone et al. 2012). Often, a diagnosis of FND is only considered when investigations are inconclusive and medical management fails, rather than being made using positive, “rule‐in” clinical signs of FND (Aybek and Perez, 2022; Espay et al. 2018).

People with FND, including those with brain tumors, may present acutely to the local Emergency department and be admitted for investigation (Stephen et al. 2021). A concern is that these admissions may result in inappropriate medical interventions (Forster et al. 2012) or treatment (Duncan et al. 2014).

The consequences of acute FND in people with brain and meningeal tumors have received little attention. In particular, the ramifications of acute FND on tumor management have not been explored. To address these issues, we focused on brain tumor patients who presented acutely with an FND that necessitated expedited investigations. Commonly, this was because the FND symptoms resembled complications associated with the tumor that required prompt action, for example, tumor progression; major side effects of tumor treatment; or development of disabling, tumor‐associated conditions, such as stroke. We complemented our case series with a systematic review of the literature on the co‐occurrence of FND in people with brain and meningeal tumors.

## 2 Materials and Methods

### 2.1 Case Series

We performed a retrospective case review of adult patients (≥18 years old) attending a regional neuro‐oncology service from mid‐2017 to mid‐2021 who presented acutely with symptoms requiring expedited investigations that were later attributed to FND. The diagnosis of FND was made in accordance with the DSM‐5 text revision (American Psychiatric Association 2022) by a Consultant Neurologist working in the neuro‐oncology service and was based on history, clinical examination emphasizing positive signs of FND (Aybek and Perez 2022; Espay et al. 2018), investigation results, and confirmation through patient follow‐up. Positive signs of FND, such as Hoover's sign, are defined in DSM‐5 under Conversion Disorder (functional neurological symptom disorder) criterion B as, “Clinical findings provide evidence of incompatibility between the symptom and recognized neurological or medical conditions” (American Psychiatric Association 2022), and have been reviewed recently (Aybek and Perez 2022).

Clinical data were obtained by reviewing all note types available within the electronic patient records of King's College Hospital and included history, clinical examination findings, brain tumor type, diagnostic investigations, psychiatric co‐morbidities and adverse life events, therapeutic interventions for FND, and outcomes (Table 1).

**TABLE 1:** Neuro‐oncology patients presenting acutely with FND.

FND onset	Tumor/WHO grade/location	FND/time to diagnosis	Expedited investigations prior to FND diagnosis	Psychiatric co‐morbidities	Surgical procedure	Adverse life events	AED stopped	Management/outcomes
Before tumor diagnosis	Glioma Low grade Right superior frontal gyrus	FSz 6 months	MRI brain EEG No epileptiform discharges	Unspecified psychiatric disorder diagnosed before tumor	Nil	NR	Not prior to patient moving back to home area	Neuropsychiatry referral declined Moved back to home area
	Meningioma Left middle cranial fossa	Attacks of left hemiparesis 12 months	CT brain CT angiogram Echocardiogram, 24 h tape EEG VT, habitual attacks not captured	Nil recorded	Tumor surgery	No	AED not started	Initial diagnosis stroke, treated with thrombolysis Attacks abated spontaneously
	Rosai–Dorfman disease Right parietal convexity	Right hemiparesis 8 months	MRI brain	Nil recorded	Nil	NR	NR	Hemiparesis improved Moved to another area
Between diagnosis and start of tumor treatment	Meningiomas WHO grade 1 Right sphenoid wing and left cavernous sinus	FSz 29 months	EEG VT, no epileptic activity during habitual attack	Nil recorded, neuropsychiatry input declined	Tumor surgery	NR	Yes	Referred for epilepsy surgery FSz diagnosed with VT Neuropsychiatry input declined
	Astrocytoma WHO grade 2 Left anterior insula	Speech disorder 2 weeks	MRI brain, EEG, no epileptic activity	Anxiety, depression diagnosed after FND started	Tumor surgery	Yes	NR	Psychiatry Venlafaxine Speech therapy, speech returned to normal <3 months No recurrence
	Cystic glioneuronal tumor Left hippocampus	Aphasia and burning mouth 5 months	CT angiogram brain, carotid and aortic arch DSA	Fibromyalgia, before tumor diagnosis	Nil	NR	NR	Initial diagnosis stroke, treated with thrombolysis No further attacks of aphasia
After tumor treatment	Astrocytoma WHO grade 3 Left thalamus and left midbrain	FSz Time to diagnosis not available	MRI brain EEG, no epileptic activity VT, no epileptic activity during habitual attack	Anxiety, depression diagnosed after tumor diagnosis and before FSz	Right sided VP shunt	NR	NR	Mirtazepine Psychology input at hospice
	Meningioma WHO grade 2 Left parafalcine	FSz 22 months	EEG, no epileptic activity during habitual attack VT, no epileptiform activity during habitual attacks	Complex PTSD diagnosed after FSz started	Tumor surgery	Yes	Yes	Neuropsychiatry CBT, FSz abated, No FSz when meningioma recurred
	Oligodendroglioma WHO grade 2 Left insula and inferior frontal cortex	FSz 4 months	MRI brain EEG VT, no epileptiform discharges during habitual attacks	Anxiety after tumor diagnosis and before FSz	Tumor surgery	No	AED not started	Neuropsychiatry CBT FSz less frequent
	Gliosarcoma WHO grade 4 Right temporal lobe	FSz Time to diagnosis not available	MRI brain VT, no epileptic discharges during habitual attack	Nil recorded	Tumor surgery	Yes	Yes	Attacks abated sufficiently for chemotherapy to restart

*Note*: Time to diagnosis represents the time from clinical presentation of FND to FND diagnosis.

Abbreviations: AED, anti‐epileptic drugs; CBT, cognitive–behavioral therapy; DSA, digital subtraction angiogram; EEG, electroencephalogram; FSz, functional seizures; NR, not recorded; PTSD, posttraumatic stress disorder; VT, videotelemetry; VP, ventriculoperitoneal.

### 2.2 Systematic Review

A systematic literature review was performed to enable comparison of our study with the literature and to facilitate recommendations from the study. The systematic review was registered with PROSPERO 2021 CRD42021250037 and is available from: https://www.crd.york.ac.uk/prospero/display_record.php?ID=CRD42021250037). The search was initially performed from April 2017 to 2021 but was then extended to May 2025.

Inclusion criteria were articles published in English peer‐reviewed journals on adults (age ≥18 years) who had either brain tumors or meningeal tumors and also had FND. All study types were included regardless of size. We searched PubMed, EMBASE, PsychINFO, Web of Science, and Cochrane Library databases for studies published in English between 1943 and May 2025 using the search strategy: (Brain neoplasms OR neuro‐oncology OR glioma OR glioblastoma OR astrocytoma OR meningioma OR schwannoma OR oligodendroglioma OR medulloblastoma OR ependymoma OR brain tumor OR brain tumor OR brain cancer OR brain neoplasia) AND (“conversion disorder” OR psychogenic OR “non epileptic” OR hysteria OR “functional neurological disorder” OR “functional disorder” OR “functional movement” OR “functional motor” OR “somatization” OR “non‐epileptic attack disorder” OR “NEAD” OR “PNES”). Articles were excluded if the full text could not be obtained or if physical symptoms were attributed to an underlying psychiatric disorder, not FND (see Supporting Information for excluded studies). The last database searches were completed on July 3, 2025.

Articles identified from electronic searches were downloaded into reference management software. Duplicated articles were removed. The articles were screened independently by two reviewers (S.C.I., D.K.J.). Discrepancies over whether an article met the study criteria were resolved by a third reviewer (G.T.F.). If the abstract met the study criteria, the full text was scrutinized by both reviewers for the following information: study design; participant demographics; presenting functional neurological symptoms; onset of functional neurological symptoms with respect to tumor diagnosis and treatment, that is, before the tumor was first diagnosed, after first diagnosis and before treatment had begun, or after tumor treatment had begun; FND diagnostic investigations; time to diagnosis; presence or absence of psychiatric co‐morbidities and significant adverse life events; and FND management and outcomes. All papers were characterized with the quality assessment tool by Murad et al. (2018) (Tables 2 and S1).

**TABLE 2:** Papers reporting cases of functional neurological disorders in neuro‐oncology patients.

Study No. patients Quality assessment (≤6)	Study design	F:M ratio	Brain/meningeal tumor type and location	FND type	Onset of FND	Prior adversity or psychiatric diagnosis	Investigations	FND treatment and treatment outcome
Reuber et al. (2002) *N* = 9 Score: 5	Case series	6:3	Meningioma: R sphenoid, L sphenoid, R frontoparietal, midline x2, L temporal Papilloma, R lat.ventricle Oligodendroglioma, L frontal Astrocytoma, R frontal	FSz	After tumor treatment	Although it was not specified if the patients had a psychiatric history prior to brain tumor diagnosis, five had a significant preoperative psychiatric history.	EEG, video EEG	Referred to psychology, anti‐epileptics withdrawn Outcome: Not specified
Garcia et al. (2018) *N* = 4 Score: 6	Case series	3:1	Oligodendroglioma, L frontal x2 Astrocytoma, R frontal Lung metastasis to brain	FSz	After tumor treatment	Not specified	EEG, video EEG, and MRI	Counselling x2 Venlafaxine x1 Methylphenidate x1 All partial response
Asadi‐Pooya and Homayoun (2020) *N* = 8 Score: 3	Retrospective cohort study	Not stated	Not specified	FSz	Not specified	History of abuse recorded, but not quantified for brain tumor patients	Video EEG, interictal EEG, and MRI	Not specified
Sumangala et al. (2022) *N* = 9 Score: 6	Case series	6:3	Oligodendroglioma L frontal x2, R frontal Astrocytoma: L frontal x2; L fronto‐temporal x1; multi‐focal x1, chiasm x1 Multinodular and Vacuolating neuronal tumor, R parietal.	FSz	*N* = 5 after tumor surgery *N* = 4 not specified	Six cases of anxiety or depression	Clinical diagnosis or EEG depending on the case	Diagnosis explanation, referral to psychology, and three patients received antidepressant medications Outcome: Reduced FSz frequency
Loebenstein et al. (2025) *N* = 6 Score: 6	Case series	4:2	Glioma: R frontal LGG: R frontal; L insula Glioblastoma: L frontal Meningioma: L frontal Lung metastasis: bilateral	FSz	After tumor treatment	Anxiety disorder NOS, panic disorder, anxiety disorder	Video EEG	“Psychological therapy,” multidisciplinary rehabilitation, “supportive care unit”
Pollak et al. (1996) *N* = 1 Score: 0	Case report	0:1	Choroid plexus papilloma, fourth ventricle	Slurred speech, ataxia, mild cognitive slowing	After tumor treatment	Previous diagnosis of schizoaffective disorder	CT imaging and clinical diagnosis	Unspecified therapeutic trials Outcome: No symptom resolution
Khu et al. (2010) *N* = 1 Score: 4	Case report	1:0	Astrocytoma, L frontal	Motor disorder affecting limb	After tumor treatment	Depression, suicidal ideation, dysfunctional home environment	CT, MRI, EMG, motor evoked potentials	Counselling and physiotherapy Outcome: Full recovery
Scarella et al. (2012) *N* = 1 Score: 4	Case report	0:1	Hypothalamic Hamartoma Focal cortical dysplasia	FSz	Tumor found incidentally, FSz 7 years later	Not stated	Video EEG and MRI	Not specified Outcome: Not specified
Young and Stoker (2012) *N* = 1 Score: 5	Case report	1:0	Meningioma, L convexity	FSz	After tumor treatment	Not stated	Standard EEG	Cognitive–behavioral therapy started on SSRIs Outcome: Less fits
Szota et al. (2013) *N* = 1 Score: 4	Case report	1:0	Oligodendroglioma, L occipital	Torticollis, formication upper limbs, and face	After tumor treatment	Severe depression	Cognitive assessments,	Behavioral therapy and SSRIs Outcome: Reduced symptoms
Slocum et al. (2017) *N* = 1 Score: 4	Case report	0:1	Astrocytoma, R frontoparietal	FSz	After tumor diagnosis	Not stated	None, based on clinical diagnosis	Underwent narrative medicine Outcome: Improved seizure control
Hebb et al. (2022) *N* = 1 Score: 5	Case report	1:0	Astrocytoma, L frontal	Functional gait disorder	Before tumor diagnosis	History of anxiety and depression as well as adverse life events	MRI and lumbar puncture	Physiotherapy, occupational therapy, and psychotherapy Outcome: Near full recovery, ongoing fatigue

Abbreviations: LGG, lower‐grade glioma; EMG, electromyography; FSz, functional seizures; L, left; NOS, not otherwise specified; R, right; SSRI, selective serotonin reuptake inhibitor.

Primary outcomes were clinical symptoms at presentation of the FND and time of FND diagnosis, divided into: before tumor diagnosis; between tumor diagnosis and start of the first round of tumor treatment; and after the first round of treatment had started. Secondary outcomes included any evidence of psychological co‐morbidities or adverse life events prior to the neuro‐oncology diagnosis. If the neuro‐oncology lesion was managed conservatively—that is, with no neurosurgical or radio‐chemotherapy intervention, then tumor management was deemed to have started at the time of diagnosis.

## 3 Results

### 3.1 Case Series

We identified 10 adult patients (median age, 46 years) who met the inclusion criteria (Table 1). Four patients had a lesion involving their right cerebral hemisphere, and six patients had a lesion involving their left cerebral hemisphere. Seven of 10 patients had either a lower‐grade glioma (*n* = 5) or a meningioma WHO grade 1 (*n* = 2) (Table 1). The three remaining patients had either a gliosarcoma, a meningioma (WHO grade 2), or Rosai–Dorfman disease (proliferation of histiocytes, which can involve the dura and mimic a meningioma).

#### 3.1.1 Acute FND in Neuro‐Oncology Patients

Functional seizures (also known as psychogenic nonepileptic seizures, dissociative seizures, or nonepileptic attack disorder) (Hingray et al. 2025) were the most common acute FND presentations, occurring in six patients. Hemiparesis developed in two patients (one right‐sided, one left‐sided). In both cases, the hemiparesis was ipsilateral to the tumor. Two patients presented with a functional speech disorder, which manifested as either a new onset stutter or intermittent episodes of speech arrest combined with burning mouth syndrome (Table 1).

Tumor‐associated seizures are a common symptom of brain tumors. One of the six patients with functional seizures had tumor‐associated seizures prior to the tumor diagnosis being established. The semiology of the functional seizures (left‐sided, focal motor) was different from the tumor‐associated seizure (olfactory). The remaining five patients had de novo functional seizures.

#### 3.1.2 Timing of Acute FND

We investigated when acute FND presented in neuro‐oncology patients. Seven of 10 patients developed acute FND (functional seizures, *n* = 3; functional hemiparesis, *n* = 2; functional speech disorders, *n* = 2) in the interval between the start of their neurological investigations and 6 weeks after the start of their first round of tumor treatment.

We explored the timing of the acute FND further by dividing the tumor illness into three periods: before diagnosis, between diagnosis and tumor treatment starting, and after treatment had started (Table 1). Functional neurological symptom onset occurred before tumor diagnosis in three patients (30%). Two of these three patients developed a functional hemiparesis during the period when they were having neurological investigations leading up to a tumor diagnosis (Table 1). The remaining patient initially presented with functional seizures on the background of a psychiatric disorder and was later (>1 year) found to have a brain tumor. An acute FND developed in three patients (30%) in the interval between tumor diagnosis and treatment (functional seizure, *n* = 1; functional speech disorder, *n* = 2). The remaining four patients (40%) all developed functional seizures after treatment had started with varying delays (<1 week, *n* = 1; 6 weeks, *n* = 1; >1 year, *n* = 2). We concluded that acute FND could occur at any stage of the tumor illness journey. However, most patients developed acute FND in the period between tumor investigations and 6 weeks after starting treatment.

#### 3.1.3 Risk Factors for Acute FND

We looked at possible risk factors for neuro‐oncology patients developing an acute FND (Espay et al. 2018; Keynejad et al. 2019; Ludwig et al. 2018). Two patients (20%) had a psychiatric diagnosis before their tumor was identified. New psychiatric diagnoses were made: in two patients (20%) during the interval between tumor diagnosis and onset of their FND, and in two patients (20%) after their tumor had been diagnosed and their FND had started (Table 1). Serious adverse life events (mental, physical, and sexual abuse; exposure to crime; close relative terminal illness or bereavement) were described by four patients. One patient reported no adverse life events. Information was not available for the remaining five patients.

#### 3.1.4 Consequences of Acute FND for Neuro‐Oncology Patients

We then examined the consequences for neuro‐oncology patients of an acute FND. The median time to diagnosis of acute FNDs from their clinical presentation was 8 months (range: 0.5–29 months). In the period between clinical presentation and diagnosis of the FND, two patients (one with functional hemiparesis, one with functional speech disorder) were initially diagnosed as strokes and received intravenous thrombolysis, and one patient with functional seizures, which were treated as tumor‐associated seizures, was referred for epilepsy surgery (Table 1).

The time from an FND diagnosis to neuropsychiatric review ranged from 3 to 28 months. There was a further delay for treatment: cognitive behavioral therapy for functional seizures started 7 months later on average. These delays arose for multiple reasons: long waiting list for neuropsychiatry referrals; the COVID‐19 pandemic; and, on occasions, due to the reluctance of patients to have a neuropsychiatric review.

Treatment of acute FND symptoms using psychiatric, psychological, and speech therapies was associated with progressive improvements in FND symptoms over several months in seven (70%) patients. The three patients who did not show improvement in their FND symptoms all had functional seizures and a comorbid psychiatric disorder.

Tumor treatment continued in parallel with management of the acute FND. However, neuro‐oncology care was interrupted for at least 1 month in three patients (30%) by delaying neurosurgery in one patient (example case history) or by disrupting subsequent oncology treatment in two patients. Tumor treatment restarted when the FND symptoms had been explained to the patient and the FND symptoms had either abated (example case history) or had improved to the point that they could be managed in parallel with tumor treatment. Precise measurement of the treatment delay was hampered by the COVID‐19 pandemic, which prolonged treatment delays in some patients.

Six patients who presented with functional seizures were on high doses of anticonvulsant medication. The dose of anticonvulsant medication was markedly reduced or stopped in three of those patients (3/6, 50%) after functional seizures were diagnosed.

We explored the longer‐lasting consequences of acute FND by reviewing patients’ follow‐up over the 4‐year study period. One patient died 1 year after presenting with functional seizures, and three patients (functional seizures, *n* = 2; functional hemiparesis, *n* = 1) left the area. At the time of leaving the area, the functional seizures in the two affected patients had not improved, whereas the functional hemiparesis had resolved. Of the six remaining patients, three (two with functional speech disorder, one with functional seizures) have had no further FND symptoms. One of these patients has had a second round of tumor treatment without a recurrence of their FND. Three patients have persistent FND, but the FND symptoms were much less disabling than at presentation in two of those patients. One patient showed no improvement in their functional seizures.

#### 3.1.5 Example Case History: Acute FND Delays Glioma Treatment

**FIGURE 1:**
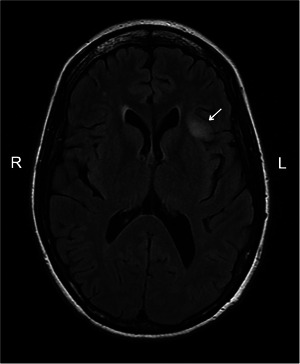
Low‐grade glioma imaged with an axial fluid‐attenuated inversion recovery (FLAIR) MRI scan. White arrow points at the low‐grade glioma (high FLAIR signal) in the left anterior insula. L, left; R, right.

An adult patient developed daily attacks of non‐pulsatile tinnitus. Otological and audiological investigations were normal. An MRI brain scan revealed a 15‐mm‐diameter, nonenhancing lesion in the left anterior insula (Figure 1). The lesion showed no restricted diffusion or increased perfusion. Interval scans showed a small increase in tumor size. The patient opted for surgical resection of the tumor. The patient needed to be woken during the operation to test their speech. In the weeks prior to the planned operation date, the patient developed a stutter. It was most pronounced when starting to speak. The patient was able to repeat some labial sounds, for example, m, m, m, but not p, p, p. However, they could not say words, such as Mandarin, that began with a labial sound, m. There was no articulatory groping. Cranial nerve examination was normal. The MRI showed no new changes. An EEG recorded no epileptiform discharges. A diagnosis of a functional stutter was made. The patient was referred to speech and language therapists and neuropsychiatrists for treatment. The patient's speech returned to normal over several weeks. The tumor surgery was delayed for 5 months, but was performed successfully with intraoperative speech testing. The neuropathological tumor diagnosis was astrocytoma, IDH1 mutant, CNS WHO grade 2. The patient made a good recovery from the tumor surgery and returned to work.

### 3.2 Systematic Review

**FIGURE 2:**
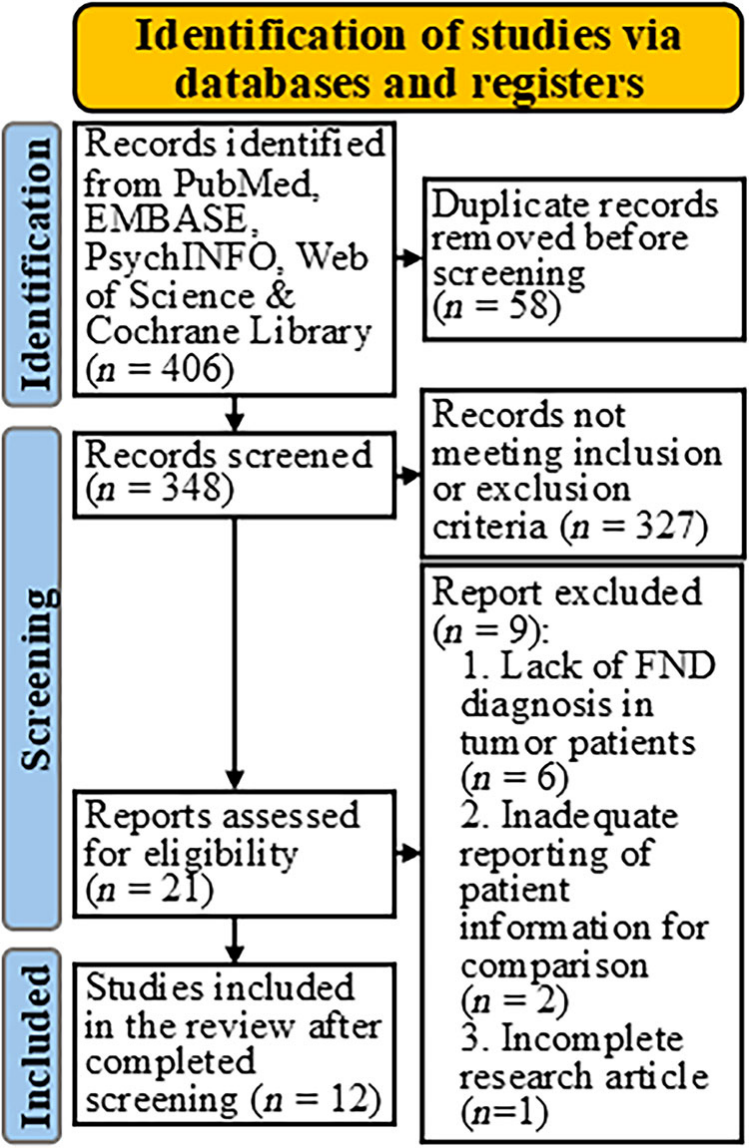
PRISMA flow diagram for systematic review. PRISMA, Preferred Reporting Items for Systematic reviews and Meta‐Analyses (see Supporting Information for excluded references).

We identified 12 papers reporting a total of 43 patients that met our review criteria (Figure 2). Five papers were case series, and seven papers reported a single case (Tables 2 and S2). Outcome data for the FNDs were not reported in two case series and two case reports (19/43 patients; Table 2). There were no systematic reviews, clinical trials, intervention studies, or case–control studies of FND in neuro‐oncology patients.

The five case series that we found were all retrospective studies of patients who developed functional seizures (Table 2) (Asadi‐Pooya and Homayoun 2020; Garcia et al. 2018; Loebenstein et al. 2025; Reuber et al., 2002; Sumangala et al. 2022). The case reports comprised three patients with functional seizures (Scarella et al. 2012; Slocum et al. 2017; Young and Stoker, 2012) and four patients with motor symptoms (slurred speech and ataxia [Pollak et al. 1996]; leg paresis [Khu et al. 2010]; torticollis [Szota et al. 2013]; functional gait disorder [Hebb et al. 2022]).

**FIGURE 3:**
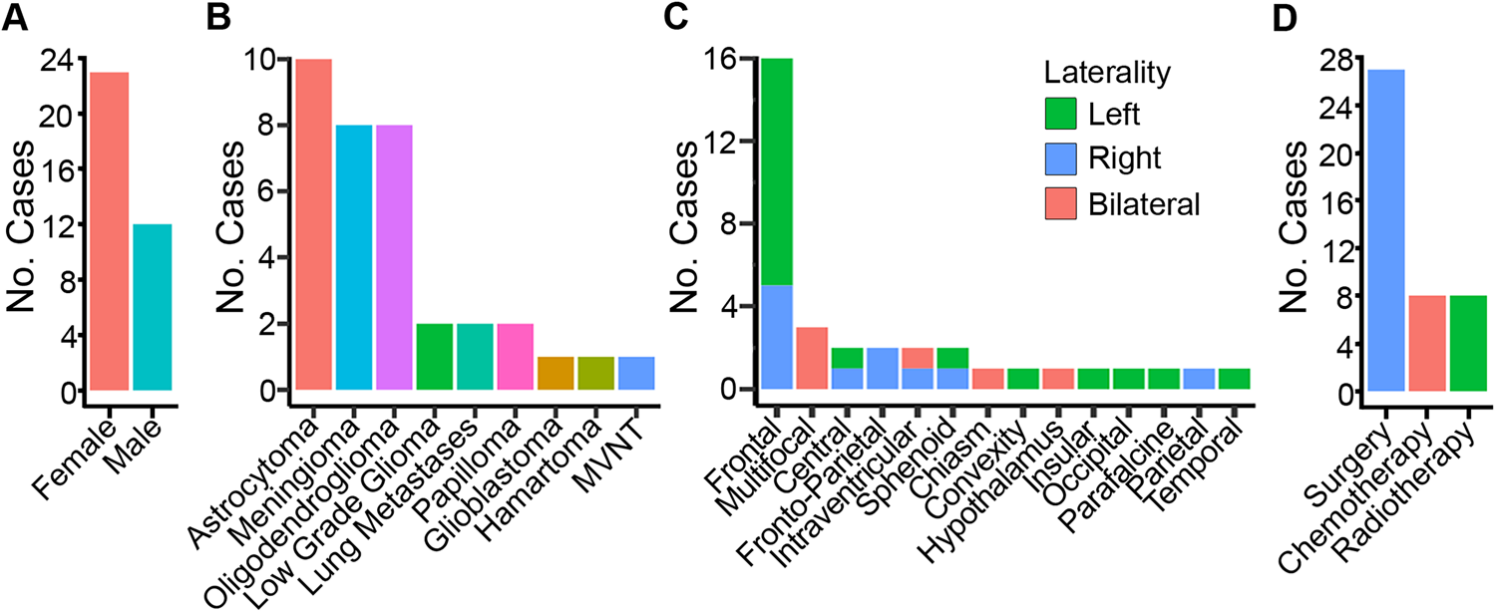
Tumor type, location, and treatment of patients reported in the systematic review. (A) Sex of patients. (B) Type of brain tumor. MVNT, multinodular and vacuolated neuronal tumor. (C) Location of brain tumor. (D) Neuro‐oncology treatment.

The patient's sex and type of tumor were specified in 35 patients (Figure 3A,B). The predominant tumor types were lower‐grade gliomas (*n* = 10 astrocytomas, *n* = 8 oligodendrogliomas, *n* = 2 lower‐grade gliomas not specified) and meningiomas (*n* = 8 patients). The remaining seven patients had five different types of tumors, including two cases of brain metastasis (Table 2; Figure 3B). A tumor location was recorded for 35 patients: left hemisphere, 18 patients; right hemisphere, 11 patients; midline, four patients; and bilateral in two patients (Figure 3C). Some details about tumor treatment were available for 32 patients (Figure 3D).

Forty‐two of 43 patients in the systematic review developed FND symptoms after tumor treatment started or after a decision was made to manage the tumor conservatively (no surgery or radio‐chemotherapy). One case report described a patient with functional symptoms before their tumor diagnosis (Hebb et al. 2022). The onset of the FND symptoms posttreatment was recorded in three of the five case series and in three case reports. The median durations of tumor treatment to onset of FND symptoms in the case series were 4 months (Sumangala et al. 2022), 12 months (Reuber et al. 2002), and 13.5 months (Garcia et al. 2018), and in the case reports were <1 day (Khu et al. 2010), <24 months (Szota et al. 2013), and >24 months (Young and Stoker, 2012). The earliest onset of FND symptoms posttreatment that we found in the literature was a case report of a patient who awoke with functional limb paresis on the day of their brain tumor surgery (Khu et al. 2010). The FND symptoms of seven patients in the systematic review started within 1 month of their tumor treatment beginning.

Thirty‐seven of 43 patients had neuroimaging, either computed tomography or MRI (Table 2). All patients with functional seizures also had an interictal EEG and/or video telemetry. Limb weakness was investigated with nerve conduction studies, electromyography, and motor evoked potentials.

FNDs were managed predominantly with psychological treatments, including counselling, behavioral therapy (unspecified), education to identify underlying stressors, and other psychological therapy approaches, such as narrative medicine and music therapy (Table 2). Functional limb weakness was also treated with physiotherapy.

A psychiatric diagnosis had been made in 17 patients (depression and/or anxiety, *n* = 11; schizoaffective disorder, *n* = 1; unspecified, *n* = 5) either before the tumor diagnosis or during tumor treatment (Table 2). No psychiatric diagnosis was recorded for 26 patients. Serious adverse life events prior to the tumor diagnosis were not routinely reported. This information was recorded in two case reports and was asked about in one case series, but no breakdown was given for the brain tumor patients (Table 2).

## 4 Discussion

Our case series focused on people with brain or meningeal tumors who developed a comorbid acute FND that negatively impacted their tumor treatment and required expedited investigations. Acute FNDs not only cause distressing symptoms in their own right but also have wider consequences for neuro‐oncology patients. We found that FND could present acutely in neuro‐oncology patients at any stage of the tumor illness. The symptoms of acute FND predominantly comprised functional seizures, functional hemiparesis, and functional speech disorders. A failure to diagnose and manage FND rapidly can result in inappropriate medical interventions and delays to tumor management. This is a problem when tumor treatment must occur within specific time limits. Finally, acute FND symptoms often became chronic.

Acute FNDs were common in the interval that straddled diagnostic tumor investigations through to the period just after tumor treatment had started. Stress is a risk factor for FND (Keynejad et al. 2019; Ludwig et al. 2018) and may be a contributing factor, as neuro‐oncology patients suffer acute stress during their tumor investigations and treatment (Goebel et al. 2012). Four of our patients had new psychiatric diagnoses made in the interval between tumor diagnosis and the onset of their acute FND.

We found that acute FNDs could lead to unnecessary interventions, such as thrombolysis, and inappropriate treatment comprising overuse of anticonvulsant medication. Similar issues have been reported in people with acute FND who do not have tumors (Duncan et al. 2014; Forster et al. 2012). Diagnosis and management of functional seizures require care because they can coexist with tumor‐associated seizures in neuro‐oncology patients and lead to escalating doses of anticonvulsants. One of our patients with undiagnosed functional seizures was referred to epilepsy surgery because of a failure to control their attacks with antiseizure medication. A correct diagnosis of functional seizures enabled us to reduce or stop anticonvulsants in 50% of patients.

Acute FND symptoms persisted for longer than 6 months in most of our patients. The prognosis of FND symptoms is mixed, with some patients doing well (Duncan et al. 2011; Simhan et al. 2020) and others poorly (Gelauff et al. 2014). Neuro‐oncology patients often undergo multiple rounds of tumor treatment and, hence, remain at high risk of persistent or recurrent FND symptoms. Treatment that puts FND symptoms into remission may prevent symptom recurrence during future treatment cycles: this was true for one of our patients. More work is needed to delineate the longer‐term effects of acute FND on the quality of life of neuro‐oncology patients.

People with lower‐grade brain tumors and meningiomas were more at risk of their tumor treatment being affected. A contributing factor may be that more aggressive tumors, such as glioblastoma, have a shorter interval between diagnosis and treatment compared with lower‐grade gliomas and meningiomas.

A larger, prospective study investigating rates of FND with different tumor locations would be needed to establish whether brain tumors increase the risk of acute FND by disrupting specific neural circuits (Perez et al. 2017) and/or through the stress associated with a neuro‐oncology diagnosis (Espay et al. 2018; Goebel et al. 2012; Keynejad et al. 2019; Ludwig et al. 2018). Such a study would benefit from combining structural and functional neuroimaging, which is usually only performed in neuro‐oncology patients when the location of speech centers needs to be identified. The patient cohort needs to be large enough to dissect the roles of the different brain networks—for example, salience, attentional, sensorimotor, right temporoparietal junction, motor planning, and limbic (Perez et al., 2021)—that have been implicated in FNDs while simultaneously addressing other possible risk factors for FNDs. These risk factors include prior adverse life events, comorbid psychiatric illness, and physical symptoms, such as fatigue and pain (Perez et al. 2021).

The presentations of acute FND in our case series differ from the systematic review in the timing and frequency of FND types found in neuro‐oncology patients. This suggests that acute FND prior to tumor treatment is an underrecognized problem.

The neurological presentations of FND that we describe are similar to those reported in the FND literature for nontumor patients (Aybek and Perez, 2022; Espay et al. 2018). Similarly, our finding that the semiology of functional seizures could be different from tumor‐associated seizures in individual patients who had both functional seizures and tumor‐associated seizures has been described previously (Garcia et al. 2018; Reuber et al. 2002; Sumangala et al. 2022). However, the literature on FND in brain tumor patients is dominated by reports of functional seizures. This may be attributable to a selection bias in the literature for functional seizures, as they are a form of FND that is well recognized due to differences in semiology, and video EEG can confirm the diagnosis.

Diagnosing FND and explaining the condition carefully is sufficient in some patients to ameliorate the FND symptoms (Duncan et al. 2011) and enable tumor treatment to restart. This emphasizes the importance of early diagnosis and explanation of FND in management. However, other patients need more intensive intervention (Example case history) to minimize delays to tumor treatment and to prevent acute FND symptoms from becoming chronic.

Treatment of comorbid FND has multiple goals, including alleviating FND symptoms to improve quality of life, preventing acute FND symptoms from becoming chronic, minimizing delays to neuro‐oncology treatment, and avoiding iatrogenic harm from unnecessary treatments.

We found delays in the diagnosis and management of acute FND in neuro‐oncology patients. Neurologists, who are often the first point of contact when medical management fails (Gilmour et al. 2020), need to be more aware that new neurological symptoms may be due to FND. Once FND has been diagnosed, neuro‐oncology patients would benefit from a dedicated multidisciplinary FND care pathway to expedite FND treatment and to prevent delays to tumor treatment. The care pathway needs to include neuropsychiatry (Espay et al. 2018), physiotherapy (Nielsen et al. 2015), speech and language therapy (Baker et al. 2021), and psychological treatment (Sharpe et al. 2011), and be integrated with existing cancer care services, including cancer psychologists and therapists.

Neuro‐oncology patients can develop acute FNDs that not only disable the patient but also delay their tumor management. Greater awareness of this possibility will increase the speed with which the correct diagnosis is made and reduce the risk of inappropriate treatment. An expedited tumor–FND care pathway is needed to optimize treatment of the FND while minimizing consequences for brain tumor management.

## Author Contributions

G.T.F. conceived the idea for the study. Analysis was performed by S.I., D.J., and G.T.F. S.I. and G.T.F. drafted the manuscript. All authors commented on the manuscript and gave approval for publication.

## Funding

Dr. Stuart Innes, Academic Clinical Fellow (ACF‐2022‐17‐010), was funded by the NIHR for this research project. The funding bodies had no role in the conduct of the study. The views expressed in this publication are those of the authors and not necessarily those of the NIHR, NHS, or the UK Department of Health and Social Care.

## Ethics Statement

This study was approved by the King's College Hospital Research, Audit and Clinical Governance committee (ref: Neurology 11/2021). The anonymous individual in this paper has given written consent to publish their example case history and MRI scan.

## Conflicts of Interest

The authors declare no conflicts of interest.

## Supporting information




**Supplementary Material**: brb371107‐sup‐0001‐TableS1.xlsx

**Supplementary Material**: brb371107‐sup‐0002‐TableS2.xlsx

**Supplementary Material**: brb371107‐sup‐0003‐SuppMat‐References‐List‐250704.docx

## Data Availability

All data relevant to this study are included in the article.
