# Supplementary material for: Consequences of Acute Presentations of Functional Neurological Disorders in Neuro‐Oncology Patients: Case Series and Systematic Review
Source: Brain Behav. 2025 Nov 30;15(12):e71107. doi: 10.1002/brb3.71107 (PMC12665190; doi:10.1002/brb3.71107)
Supplement: Supplementary file 3 — Supplementary Material: brb371107‐sup‐0003‐SuppMat‐References‐List‐250704.docx [file BRB3-15-e71107-s003.docx]

**Supplementary References**

**Lack of FND diagnosis in tumor patients:**

1. Peters, K. and M. Johnson, Functional Neurological Disorders in Primary Brain Tumor Patients. Neurology, 2022. 98(18 SUPPL).

2. Mainio, A., et al., Somatization symptoms are related to right-hemispheric primary brain tumor: A population-based prospective study of tumor patients in northern Finland. Psychosomatics, 2009. 50(4): p. 331-335.

3. Keane, J.R., Hysterical Hemianopia: The ‘Missing Half’ Field Defect. Archives of Ophthalmology, 1979. 97(5): p. 865-866.

4. Harris, J., Depression and hysteria as symptoms of brain tumor. Henry Ford Hospital Medical Journal, 1965. 13(4): p. 457-459.

5. Anand, S., et al., Why do neurologists miss catatonia in neurology emergency? A case series and brief literature review. Clinical Neurology and Neurosurgery, 2019. 184.

6. Taga, A., et al., Meningeal Dissemination and Drop Metastasis From Glioma Presenting With Non-Epileptic Myoclonus and Minipolymyoclonus. The Neurohospitalist, 2024.

Inadequate reporting of patient information for comparison:

7. Kaplan, C.P., SCL 90-R interpretation and brain tumour: A correction factor? *Brain Injury*, 1998. **12**(11): p. 977-985.

8. Kaplan, C.P., et al., Interpretive risks: the use of the Hopkins Symptom Checklist 90 Revised (SCL 90-R) with brain tumour patients. Brain Injury, 1998. **12**(3): p. 199-205.

Incomplete research article:

9. Gogela, L.J. and C.W. Rucker, Psychogenic changes in the field of vision associated with tumors of the frontal lobe of the brain. *American Journal of Ophthalmology*, 1951. **34**(2-1): p. 185-188.
